# Relationship between Oral Hypofunction and Sarcopenia in Community-Dwelling Older Adults: The Otassha Study

**DOI:** 10.3390/ijerph18126666

**Published:** 2021-06-21

**Authors:** Yoshihiro Kugimiya, Masanori Iwasaki, Yuki Ohara, Keiko Motokawa, Ayako Edahiro, Maki Shirobe, Yutaka Watanabe, Shuichi Obuchi, Hisashi Kawai, Yoshinori Fujiwara, Kazushige Ihara, Hunkyung Kim, Takayuki Ueda, Hirohiko Hirano

**Affiliations:** 1 Department of Removable Prosthodontics and Gerodontology, Tokyo Dental College, Tokyo 101-0061, Japan; uedat@tdc.ac.jp; 2 Research Team for Promoting Independence and Mental Health, Tokyo Metropolitan Institute of Gerontology, Tokyo 173-0015, Japan; iwasaki@tmig.or.jp (M.I.); yohara@tmig.or.jp (Y.O.); kikiki_1004@yahoo.co.jp (K.M.); aedahiro514@gmail.com (A.E.); ywata@den.hokudai.ac.jp (Y.W.); kimhk@tmig.or.jp (H.K.); h-hiro@gd5.so-net.ne.jp (H.H.); 3 The Tokyo Metropolitan Support Center for Preventative Long-Term and Frail Elderly Care, Tokyo Metropolitan Institute of Gerontology, Tokyo 173-0015, Japan; mashirobe@gmail.com; 4 Gerodontology, Department of Oral Health Science, Faculty of Dental Medicine, Hokkaido University, Sapporo 060-8586, Japan; 5 Research Team for Human Care, Tokyo Metropolitan Institute of Gerontology, Tokyo 173-0015, Japan; obuchipc@tmig.or.jp (S.O.); hkawai@tmig.or.jp (H.K.); 6 Research Team for Social Participation and Community Health, Tokyo Metropolitan Institute of Gerontology, Tokyo 173-0015, Japan; fujiwayo@tmig.or.jp; 7 Department of Social Medicine, Hirosaki University School of Medicine, Aomori 036-8562, Japan; ihara@hirosaki-u.ac.jp; 8 Tokyo Metropolitan Geriatric Hospital, Tokyo 173-0015, Japan

**Keywords:** oral hypofunction, sarcopenia, oral function, physical health, older adults, Japan

## Abstract

Oral hypofunction, resulting from a combined decrease in multiple oral functions, may affect systemic-condition deterioration; however, few studies have examined the association between oral hypofunction and general health among older adults. In this cross-sectional study, we examined the relationship between oral hypofunction and sarcopenia in community-dwelling older adults. We included 878 adults (268 men and 610 women, mean age 76.5 ± 8.3 years). Tongue coating index, oral moisture, occlusal force, oral diadochokinesis (/pa/,/ta/,/ka/), tongue pressure, mas-ticatory function, and swallowing function were evaluated as indicators of oral hypofunction. Grip strength, gait speed, and skeletal muscle mass index were measured as diagnostic sarcopenia parameters. The association between oral hypofunction and sarcopenia was examined via logistic regression using sarcopenia as the dependent variable. Oral hypofunction prevalence was 50.5% overall, 40.3% in men, and 54.9% in women. The prevalence of sarcopenia was 18.6% overall, 9.7% in men, and 22.5% in women. A logistic regression showed oral hypofunction, age, body mass index, higher-level functional capacity, and serum albumin level were significantly associated with sarcopenia. Sarcopenia occurred at an increased frequency in patients diagnosed with oral hypofunction (odds ratio: 1.59, 95% confidence interval: 1.02–2.47); accordingly, oral hypofunction appears to be significantly associated with sarcopenia.

## 1. Introduction

Japan has a rapidly aging population. As the population ages, the number of people in need of nursing care, and the cost of social security benefits increase correspondingly [1]. Therefore, extending the healthy life expectancy of older adults has become an urgent issue.

In 2016, the Japanese Society of Gerodontology (JSG) recognized oral hypofunction as a new disease in the oral field [2], and it was introduced into the public insurance system in Japan in 2018 [3]. As of 2021, the public insurance system in Japan defines oral hypofunction as “a disease in which oral function is complexly reduced, not only by aging but also by various factors pertaining to diseases and disorders”. Oral hypofunction is diagnosed by assessing the presence or absence of decreased oral function in seven dimensions, i.e., poor oral hygiene, oral dryness, reduced occlusal force, decreased tongue-lip motor function, decreased tongue pressure, decreased masticatory function, and deterioration of swallowing function. The items examination examined have been determined to be associated with malnutrition [2,4]. Individuals with decreased levels of functionality in three or more of the seven dimensions considered are diagnosed with oral hypofunction.

Decreases in individual levels of oral function have been shown to influence general health deterioration [5,6,7,8,9]. Oral hypofunction is a factor hat influences the deterioration of general health. To date, however, few studies have examined the association between oral hypofunction and general health among older adults [10]. If the relationship between oral hypofunction and systemic diseases is clarified, it may be possible to extend the healthy life expectancy of older adults from the perspective of dentistry.

In this study, we focused on sarcopenia as an outcome of oral hypofunction, as it is a principal geriatric syndrome, and malnutrition is a risk factor for its development [11,12], as is oral hypofunction. The Asian Working Group for Sarcopenia defines sarcopenia as “age-related loss of skeletal muscle mass plus loss of muscle strength and/or reduced physical performance” [12]. Sarcopenia can have a negative impact on daily life and decrease quality of life. Therefore, the prevention of sarcopenia, and timely intervention are needed. If the relationship between oral hypofunction and sarcopenia is clarified, it may be possible to address sarcopenia from the standpoint of dental professionals. The purpose of this study was to clarify the relationship between oral hypofunction and sarcopenia among community-dwelling older adults.

## 2. Materials and Methods

### 2.1. Research Design

This cross-sectional study used data collected in 2018 for “The Otassha Study”, a cohort study that evaluated comprehensive health examination data conducted by the Tokyo Metropolitan Institute of Gerontology. Data was collected from people aged 65 years or older living in Itabashi city, Tokyo. Potential participants were informed of the date, time, and venue of the health examination in advance by letter, and the health examination was conducted for those who appeared on the allotted date. The purpose and content of the health examination were explained to all participants in writing and orally, and written consent for participation in the study was obtained from all study participants. Individuals with missing survey data were excluded. All health examination evaluators received training on measurement methods and the use of evaluation instruments in advance, and evaluation criteria were standardized. This study was approved by the Ethics Committee of the Tokyo Metropolitan Institute of Gerontology (2006-17, 2011-48, 2018-Zin1, 16), and was conducted in accordance with the Strengthening the Reporting of Observational Studies in Epidemiology Statement [13].

### 2.2. Number of Teeth

Numbers of present and functional teeth were evaluated via an intra-oral examination by dentists and dental hygienists [14]. Present teeth were defined as those in which crowns had erupted, and were excluded if they were not occluded, were stump teeth, or showed significant looseness. Functional teeth included present teeth, pontics for bridges, artificial teeth for removable dentures, and superstructures for implants.

### 2.3. Oral Hypofunction

To diagnose oral hypofunction, the degree of tongue coating, oral moisture, occlusal force, tongue-lip motor function, tongue pressure, masticatory function, and swallowing function were evaluated by dentists and dental hygienists. The presence or absence of seven items including poor oral hygiene, oral dryness, reduced occlusal force, decreased tongue-lip motor function, decreased tongue pressure, decreased masticatory function, and deterioration of swallowing function were assessed, and participants who experienced decreased functionality of least three items were diagnosed with oral hypofunction based on diagnostic criteria published by the JSG in 2016 [2]. Removable denture users underwent oral function examinations with their removable dentures in place.

#### 2.3.1. Poor Oral Hygiene

Degree of tongue coating was evaluated using the tongue coating index (TCI). To determine TCI values, the tongue was divided into nine blocks, and the degree of tongue coating of each block was visually inspected [15]. Poor oral hygiene was defined as a TCI value ≥ 50% [2].

#### 2.3.2. Oral Dryness

The degree of oral moisture was assessed using an oral moisture checker (Mucus, Life Co., Ltd., Saitama, Japan) [16,17]. The device was pressed against the center of the tongue dorsum at approximately 200 gf to measure mucosal moisture content. Three successive measurements were performed, and the median oral moisture value was used. Oral dryness was defined as an oral moisture level < 27.0 [2].

#### 2.3.3. Reduced Occlusal Force

Occlusal force was measured using a pressure-sensitive sheet (Dental Prescale 50H Type-R, Fuji Film Co., Tokyo, Japan) scanned using an image scanner (Occluzer, FPD-707, Fuji Film Co.) [18,19,20]. Participants bit a 98-μm thick, pressure-sensitive sheet with maximal force at the intercuspal position for 3 s. Occlusal contact points on the pressure-sensitive sheet were read by an image scanner, and occlusal force was calculated. Participants with occlusal force values determined to be <200 N were deemed to have reduced occlusal force [2].

#### 2.3.4. Decreased Tongue–Lip Motor Function

Tongue-lip motor function was measured based on the dexterity of the tongue and lips using an automatic counter (KENKOU-KUN handy, Takei Scientific Instruments Co., Ltd., Niigata, Japan) [21]. Sounds including/pa/,/ta/, and/ka/were repeated as quickly as possible for 5 s each, and the number of syllables pronounced per s was measured to determine the oral diadochokinesis (ODK) value. Decreased tongue-lip motor function was defined as a/pa/,/ta/, or/ka/ODK value of <6.0 times/s [2].

#### 2.3.5. Decreased Tongue Pressure

Tongue pressure was assessed using a tongue pressure measurement device (JMS tongue pressure device TPM-01, JMS Co., Ltd., Hiroshima, Japan) [22,23]. To evaluate tongue pressure, the air-inflated balloon portion of a disposable oral probe was placed at the anterior section of the palate, and each study participant pressed the balloon against their palate using their tongue for 7 s. Participants compressed balloons three times with maximum voluntary effort at 30-s intervals, the mean maximum pressure recorded was used as the tongue pressure value. Participants with a tongue pressure value < 30 kPa were considered to have decreased tongue pressure [2].

#### 2.3.6. Decreased Masticatory Function

Masticatory function was evaluated using gummy jelly (test gummy jelly, UHA Mikakuto Co., Ltd., Osaka, Japan) [24,25]. Participants chewed gummy jelly 30 times, and spat the chewed material onto gauze. Chewed gummy jelly was visually compared with a score chart that defined 10 levels of masticatory function to determine values. Those who scored of ≤2 were considered to have decreased masticatory function [2].

#### 2.3.7. Deterioration of Swallowing Function

Swallowing function was assessed using a 10-item Eating Assessment Tool [26], which is a self-administered questionnaire with 10 questions that assess swallowing function using a 5-point Likert scale ranging from 0 to 4. Participants with a total score of ≥3 were considered to have deteriorated swallowing function [2].

### 2.4. Sarcopenia

To diagnose sarcopenia, parameters including grip strength, gait speed, and appendicular skeletal muscle mass of participants were measured by researchers with expertise in physical functions assessed. Using the Asian Working Group for Sarcopenia 2019 (AWGS2019) algorithm as a reference, sarcopenia was diagnosed if the participant had either low appendicular skeletal muscle mass and muscle strength levels or low appendicular skeletal muscle and low physical performance. Sarcopenia was considered severe if participants were determined to be positive for both sarcopenia indicators [12].

#### 2.4.1. Low Muscle Strength

To evaluate muscle strength, the grip strength of the dominant hand was measured using a Smedley-type hand dynamometer (Grip-A, Takei Scientific Instruments Co., Ltd., Niigata, Japan). Participants were required to grip of the hand dynamometer in a standing position with their arms lowered at a position that felt natural. Measurements were obtained twice, and the higher value was used to record handgrip strength. A handgrip strength < 28 kg and <18 kg for men and women, respectively, were defined as low muscle strength [12].

#### 2.4.2. Low Physical Performance

Gait speed was measured to evaluate physical performance. Participants were instructed to walk at a normal pace along a straight 11-m walkway with 3-m and 8-m points marked with tape on a flat floor. Time needed to walk the 5-m distance between tape marks was measured to determine gait speed [27]. A gait speed lower than 1 m/s was indicated low physical performance [12].

#### 2.4.3. Low Appendicular Skeletal Muscle Mass

Appendicular skeletal muscle mass was measured using bio-electrical impedance analysis (InBody 770, InBody Inc., Seoul, Korea). Each measured appendicular skeletal muscle mass value was divided by the square of the height (m conversion) of each participant to calculate skeletal muscle mass index (SMI). An SMI value of <7.0 kg/m^2^ and <5.7 kg/m^2^ for men and women, respectively, indicated low appendicular skeletal muscle mass [12].

### 2.5. Other Recorded Variables

Other survey items included the following: age; years of education; body mass index (BMI); drinking and smoking habits; living situation; depression assessed with a questionnaire [9]; higher-level functional capacity assessed using the Japan Science and Technology Agency index of competence (JST-IC) [28], the Japanese version of the Mini Mental State Examination (MMSE-J) for cognitive function [29,30]; medical history recorded through an interview (stroke, heart disease, diabetes, and cancer); and serum albumin and hemoglobin A1c levels.

### 2.6. Statistical Analysis

Patients with sarcopenia and severe sarcopenia were included in the sarcopenia group when performing statistical analyses. Between-group comparisons of continuous variables were performed using the Mann–Whitney U test, and categorical variables were analyzed with the chi-squared test. The association between oral hypofunction and sarcopenia was examined via logistic regression analysis using the forced entry method, with sarcopenia as the dependent variable. Inclusion of variables in the model was based on existing knowledge of risk factors for sarcopenia. Statistical analyses were performed using IBM SPSS version 27 (IBM Corp., Armonk, NY, USA). As this was a secondary study of the Otassha Study, no prior sample size calculations were performed

## 3. Results

### 3.1. Participants

**Figure 1:**
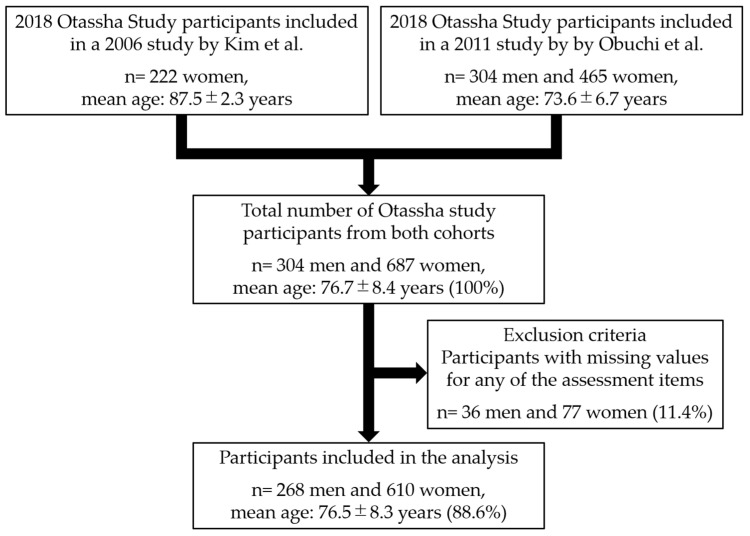
Flowchart of the study design.

A flowchart detailing the design of this study is shown in Figure 1. There were 991 adults (100%) (304 men and 687 women, mean age: 76.7 ± 8.4 years) who visited the venue and participated in the comprehensive health examination. Among the visitors, 113 (11.4%) with missing survey data were excluded from the analysis. The total number of participants included in the study was 878 adults (88.6%) (268 men and 610 women, mean age: 76.5 ± 8.3 years).

### 3.2. Characteristics of Study Participants and between Group Comparisons

Characteristics of the study population and survey item differences according to sex, and presence of sarcopenia and oral hypofunction are shown in Table 1 and Table 2. Table 1 and Table 2 contain between-group comparisons of continuous and categorical variables, respectively. The prevalence of oral hypofunction was 50.5% overall, 40.3% in men, and 54.9% in women. The prevalence of oral hypofunction in men and women significantly differed (*p* < 0.001). The prevalence of sarcopenia and severe sarcopenia was 14.4% and 4.2% overall, 7.2% and 2.2% in men, and 17.4% and 5.1% in women, respectively. The prevalence of sarcopenia (both severe and not severe) in men and women significantly differed (*p* < 0.001). When sarcopenia and severe sarcopenia were considered collectively, sarcopenia prevalence was 18.6% overall, 9.7% in men, and 22.5% in women. The prevalence of oral hypofunction between the robust not diagnosed with sarcopenia and sarcopenia groups was 45.3% and 73.0%, respectively, and the values were determined to significantly differ (*p* < 0.001). Among subordinate symptoms of oral hypofunction considered, rates of reduced occlusal force (*p* < 0.001), decreased tongue-lip motor function (*p* < 0.001), decreased tongue pressure (*p* < 0.001), decreased masticatory function (*p* < 0.001), and deterioration of swallowing function (*p* = 0.028) were significantly higher in patients of the sarcopenia versus robust group.

### 3.3. Variables Associated with Sarcopenia

Results of the multivariable logistic regression analysis in which sarcopenia was the dependent variable are shown in Table 3. Findings showed a significant association between oral hypofunction and sarcopenia. Sarcopenia frequency in the oral hypofunction group was significantly elevated (odds ratio: 1.585, 95% confidence interval: 1.019–2.465). In addition, age, BMI, JST-IC score, and serum albumin level were significantly associated with sarcopenia.

## 4. Discussion

In this study, we focused on sarcopenia [11,12] as an outcome of oral hypofunction because malnutrition is a risk factor for its development. As oral hypofunction is a new disease, associated data is limited. If general health deterioration risk due to the accumulation of decreases in oral function in old age is clarified, the knowledge will likely facilitate the implementation of planning measures aiming to extend healthy life expectancy from the perspective of dentistry.

Oral function evaluation may help confirm the effectiveness of treatment, and predict future general-health deterioration [8]. It is also known that decreased oral function is a prodromal symptom of severe diseases including neuromuscular conditions [31,32,33,34,35,36,37,38]. It is clearly important to conduct oral function examinations on a broad range of patients, since they have the potential to incidentally detect severe diseases at an early stage.

A strength of this study is that we analyzed a comprehensive set of oral-health and function variables. To the best of our knowledge, this was the first study to demonstrate that oral hypofunction, diagnosed in strict accordance with criteria published by the JSG, was associated with sarcopenia in community-dwelling older adults. One previous study reported an association between oral hypofunction and sarcopenia [39]. Although it was an important investigation, it should be noted that prior authors did not apply the diagnostic criteria officially proposed by the JSG. Specifically, masticatory ability was based on self-reports, which could have introduced bias.

In our study, the prevalence of oral hypofunction based on diagnostic criteria published by the JSG was 50.5% overall, 40.3% among men, and 54.9% among women. Previous reports have indicated that the prevalence of oral hypofunction among older adults living in different regions of Japan is 43.6–61.6% overall, 39.0–62.6% among men, and 46.9–63.1% among women [10,39,40,41]. The prevalence of oral hypofunction in our study was within the range previously reported among community-dwelling older adults.

The prevalence of sarcopenia in our study population was 18.6% overall, 9.7% among men, and 22.5% among women. The prevalence of sarcopenia in older Asians using AWGS2019 criteria has ranged from 16.4 to 22.8% overall, 11.5 to 21.8% among men, and 16.7 to 23.1% among women [42,43,44]. The prevalence of men in the present study was lower than that of previous reports. This difference suggests that the present population likely included fewer male participants who tend to have reduced muscle mass, strength, and physical performance.

The results of the logistic regression analysis showed that age, BMI, JST-IC representing higher-level functional capacity, and serum albumin level reflecting nutritional and inflammatory status were significantly associated with sarcopenia. Age and BMI reportedly have a clear association with sarcopenia [12]. Low serum albumin levels have also been reported to be low in those with sarcopenia [45,46]. It has also been shown that higher-level functional capacity is associated with sarcopenia [47,48]. Results of the present study are in accordance with prior findings.

This study revealed a significant association between oral hypofunction and sarcopenia, even after adjusting for effects of multiple variables. Numerous studies have shown that decreased oral function leads to poor nutritional status [9,20,49]. It has also been suggested that a decrease in oral function affects the choice of food intake [50]. These reports potentially support a pathway in which the presence of various decreases in parameters of oral function promote the development of sarcopenia by worsening nutritional status by promoting an unbalanced intake of foods and nutrients. It has been reported that both prosthetic treatment and simplified dietary advice during dental treatment improve oral function and nutritional status in older adults [51,52,53]. When an older adult is found to have overlapping decreases in oral functions, addressing each affected oral function parameter and providing simplified dietary advice may help reduce sarcopenia risk. Hironaka et al. reported that a decrease in oral function may not only directly lead to physical function decline, but may also affect the decline in physical functions indirectly via a pathway mediated by declining social functions [9]. In addition to dental treatment and dietary advice, dental professionals may reduce risk of sarcopenia by providing a comprehensive response that includes addressing social function decline.

As this was a cross-sectional study, it was not possible to determine the direction of the association between oral hypofunction and sarcopenia. To date, few longitudinal studies have examined the impact of various oral function parameter decreases on sarcopenia [8]. Longitudinal studies are needed to clarify the temporal relationship between oral hypofunction and sarcopenia.

## 5. Conclusions

In this study, we found a significant association between oral hypofunction and sarcopenia among community-dwelling older adults. The prevalence of oral hypofunction based on diagnostic criteria published by the JSG in 2016 was 50.5% overall, 40.3% in men, and 54.9% in women. The prevalence of sarcopenia based on diagnostic criteria of AWGS2019 was 18.6% overall, 9.7% in men and 22.5% in women. Multidisciplinary action is needed to establish comprehensive measures for managing the oral health of patients with oral hypofunction, especially as a means to avoid future general-health deterioration.

## Figures and Tables

**Table 1:** Simple comparison of participant characteristics and continuous variables.

	Overall	Women	Men		Robust	Sarcopenia		Robust	Oral Hypofunction	
	(n = 878)	(n = 610)	(n = 268)		(n = 715)	(n = 163)		(n = 435)	(n = 443)	
Continuous Variables	Median, (Q1, Q3)	Median, (Q1, Q3)	Median, (Q1, Q3)	*p*-Value	Median, (Q1, Q3)	Median, (Q1, Q3)	*p*-Value	Median, (Q1, Q3)	Median, (Q1, Q3)	*p*-Value
Variables of oral hypofunction										
Tongue coating index (%)	22.2, (11.1, 50.0)	22.2, (11.1, 50.0)	33.3, (16.7, 61.1)	<0.001	22.2, (11.1, 50.0)	22.2, (11.1, 38.9)	0.047	16.7, (11.1, 38.9)	33.3, (11.1, 55.6)	<0.001
Oral moisture	27.1, (24.9, 28.8)	26.9, (24.8, 28.6)	27.4, (25.2, 29.3)	0.008	27.2, (25.0, 28.9)	26.6, (24.4, 28.6)	0.096	27.8, (26.3, 29.3)	26.1, (24.3, 28.0)	<0.001
Occlusal force (N)	257.1, (121.0, 417.8)	213.8, (101.8, 366.4)	367.7, (221.3, 559.3)	<0.001	293.2, (140.4, 435.9)	163, (72.8, 288.7)	<0.001	352.2, (240.0, 517.4)	155.5, (79.6, 301.2)	<0.001
Oral diadochokinesis/pa/ (time/s)	6.4, (5.8, 6.8)	6.2, (5.8, 6.8)	6.4, (6.0, 6.8)	0.002	6.4, (6.0, 6.8)	6.0, (5.4, 6.6)	<0.001	6.6, (6.2, 7.0)	6.2, (5.6, 6.6)	<0.001
Oral diadochokinesis/ta/ (time/s)	6.4, (5.8, 6.8)	6.2, (5.8, 6.8)	6.4, (6.0, 6.8)	0.004	6.4, (5.8, 6.8)	6.0, (5.4, 6.4)	<0.001	6.6, (6.2, 7.0)	6, (5.6, 6.4)	<0.001
Oral diadochokinesis/ka/ (time/s)	5.8, (5.4, 6.4)	5.8, (5.4, 6.4)	6.0, (5.2, 6.4)	0.917	6.0, (5.4, 6.4)	5.6, (5.0, 6.0)	<0.001	6.2, (5.8, 6.6)	5.6, (5.0, 6.0)	<0.001
Tongue pressure (kPa)	31.8, (26.3, 36.9)	31.3, (25.8, 35.7)	33.4, (27.8, 39)	<0.001	32.9, (27.8, 37.8)	27.2, (20.6, 31.8)	<0.001	34.6, (30.8, 39.2)	28.3, (23.5, 33.2)	<0.001
Gummy jelly score	5, (3, 6)	5, (2, 6)	6, (5, 7)	<0.001	5, (4, 6)	4, (1, 6)	<0.001	6, (5, 6)	4, (1, 6)	<0.001
EAT-10 score	1, (0, 3)	1, (0, 3)	1, (0, 4)	0.516	1, (0, 3)	1, (0, 5)	0.004	0, (0, 2)	2, (0, 5)	<0.001
Oral hypofunction score	3, (2, 4)	3, (2, 4)	2, (1, 3)	<0.001	2, (1, 3)	4, (2, 5)	<0.001	2, (1, 2)	4, (3, 4)	<0.001
Variables of sarcopenia										
Handgrip strength (kg)	24.0, (18.0, 31.0)	21.0, (17.0, 25.0)	35.0, (30.3, 40.0)	<0.001	25.0, (21.0, 33.0)	16, (14, 19)	<0.001	26, (21, 35)	21, (16, 27)	<0.001
Gait speed (m/s)	1.32, (1.11, 1.52)	1.32, (1.09, 1.47)	1.39, (1.22, 1.55)	<0.001	1.39, (1.19, 1.52)	1.09, (0.91, 1.32)	<0.001	1.39, (1.22, 1.56)	1.25, (1.04, 1.43)	<0.001
Skeletal muscle mass index (kg/m^2^)	6.1, (5.6, 7.0)	5.8, (5.3, 6.2)	7.5, (7.0, 7.9)	<0.001	6.3, (5.8, 7.3)	5.3, (4.9, 5.6)	<0.001	6.3, (5.7, 7.4)	5.9, (5.4, 6.7)	<0.001
Other recorded variables										
Age (years)	76, (68, 85)	78, (70, 86)	71, (68, 78.8)	<0.001	74, (68, 81)	86, (80, 89)	<0.001	72, (67, 79)	81, (72, 86)	<0.001
Number of present teeth	23, (14, 27)	22, (12, 26)	25, (20, 28)	<0.001	24, (17, 27)	20, (7, 25)	<0.001	26, (21, 28)	19, (8, 25)	<0.001
Number of functional teeth	28, (27, 28)	28, (27, 28)	28, (27, 28)	0.081	28, (27, 28)	28, (27, 28)	0.046	28, (27, 28)	28, (27, 28)	0.028
Body mass index (kg/m^2^)	22.5, (20.6, 24.7)	22.0, (20.2, 24.3)	23.4, (21.7, 25.2)	<0.001	22.8, (20.8, 24.9)	21.1, (18.8, 22.9)	<0.001	22.8, (20.8, 24.8)	22.3, (20.3, 24.5)	0.056
Education (years)	12, (12, 16)	12, (11, 14)	16, (12, 16)	<0.001	12, (12, 16)	12, (10, 12)	<0.001	12, (12, 16)	12, (10, 14)	<0.001
JST-IC score	12, (9, 14)	12, (9, 14)	12, (10, 14)	0.009	12, (10, 14)	9, (7, 12)	<0.001	13, (11, 14)	11, (8, 13)	<0.001
MMSE-J score	29, (28, 30)	29, (28, 30)	29, (28, 30)	0.138	29, (28, 30)	29, (27, 29)	<0.001	29, (28, 30)	29, (27, 30)	<0.001
Serum albumin (g/dL)	4.2, (4.1, 4.4)	4.3, (4.1, 4.4)	4.2, (4.1, 4.4)	0.073	4.3, (4.1, 4.4)	4.2, (4.0, 4.4)	0.001	4.3, (4.1, 4.4)	4.2, (4.1, 4.4)	0.008
Hemoglobin A1c (%)	5.7, (5.5, 6.0)	5.7, (5.5, 6.0)	5.6, (5.4, 6.0)	0.282	5.7, (5.5, 6.0)	5.6, (5.4, 6.0)	0.493	5.7, (5.5, 6.0)	5.7, (5.4, 6.0)	0.635

Abbreviations: EAT-10: 10-item Eating Assessment Tool; JST-IC: Japan Science and Technology Agency Index of Competence; MMSE-J: Japanese version of the mini mental state examination; Q1: first quartile; Q3: third quartile. Statistical analysis was performed using the Mann–Whitney U test.

**Table 2:** Simple comparison of participant characteristics and categorical variables.

		Overall	Women	Men		Robust	Sarcopenia		Robust	Oral Hypofunction	
		(n = 878)	(n = 610)	(n = 268)		(n = 715)	(n = 163)		(n = 435)	(n = 443)	
Category Variables		N, (%)	N, (%)	N, (%)	*p*-Value	N, (%)	N, (%)	*p*-Value	N, (%)	N, (%)	*p*-Value
Variables of oral hypofunction											
Poor oral hygiene		254, (28.9)	154, (25.2)	100, (37.3)	<0.001	217, (30.3)	37, (22.7)	0.052	87, (20.0)	167, (37.7)	<0.001
Oral dryness		414, (47.2)	307, (50.3)	107, (39.9)	0.004	327, (45.7)	87, (53.4)	0.078	130, (29.9)	284, (64.1)	<0.001
Reduced occlusal force		352, (40.1)	291, (47.7)	61, (22.8)	<0.001	256, (35.8)	96, (58.9)	<0.001	72, (16.6)	280, (63.2)	<0.001
Decreased tongue–lip motor function		485, (55.2)	343, (56.2)	142, (53.0)	0.373	363, (50.8)	122, (74.8)	<0.001	147, (33.8)	338, (76.3)	<0.001
Oral diadochokinesis/pa/	<6 times/s	236, (26.9)	179, (29.3)	57, (21.3)	0.013	167, (23.4)	69, (42.3)	<0.001	65, (14.9)	171, (38.6)	<0.001
Oral diadochokinesis/ta/	<6 times/s	250, (28.5)	186, (30.5)	64, (23.9)	0.046	180, (25.2)	70, (42.9)	<0.001	68, (15.6)	182, (41.1)	<0.001
Oral diadochokinesis/ka/	<6 times/s	440, (50.1)	309, (50.7)	131, (48.9)	0.628	327, (45.7)	113, (69.3)	<0.001	134, (30.8)	306, (69.1)	<0.001
Decreased tongue pressure		285, (32.5)	216, (35.4)	69, (25.7)	0.005	193, (27.0)	92, (56.4)	<0.001	62, (14.3)	223, (50.3)	<0.001
Decreased masticatory function		191, (21.8)	164, (26.9)	27, (10.1)	<0.001	123, (17.2)	68, (41.7)	<0.001	16, (3.7)	175, (39.5)	<0.001
Deterioration of swallowing function		256, (29.2)	174, (28.5)	82, (30.6)	0.534	197, (27.6)	59, (36.2)	0.028	65, (14.9)	191, (43.1)	<0.001
Oral hypofunction		443, (50.5)	335, (54.9)	108, (40.3)	<0.001	324, (45.3)	119, (73.0)	<0.001	-	-	
Variables of sarcopenia											
Low muscle strength		215, (24.5)	174, (28.5)	41, (15.3)	<0.001	77, (10.8)	138, (84.7)	<0.001	64, (14.7)	151, (34.1)	<0.001
Low physical performance		114, (13.0)	97, (15.9)	17, (6.3)	<0.001	52, (7.3)	62, (38.0)	<0.001	24, (5.5)	90, (20.3)	<0.001
Low appendicular skeletal muscle mass		348, (39.6)	279, (45.7)	69, (25.7)	<0.001	185, (25.9)	163, (100.0)	<0.001	141, (32.4)	207, (46.7)	<0.001
Sarcopenia	Robust	715, (81.4)	473, (77.5)	242, (90.3)	<0.001	715, (100.0)	0, (0)	<0.001	391, (89.9)	324, (73.1)	<0.001
	Sarcopenia	126, (14.4)	106, (17.4)	20, (7.5)		0, (0)	126, (77.3)		42, (9.7)	84, (19.0)	
	Severe sarcopenia	37, (4.2)	31, (5.1)	6, (2.2)		0, (0)	37, (22.7)		2, (0.5)	35, (7.9)	
Other recorded variables											
Sex	Women	610, (69.5)	-	-		473, (66.2)	137, (84.0)	<0.001	275, (63.2)	335, (75.6)	<0.001
Body mass index	<18.5	85, (9.7)	72, (11.8)	13, (4.9)	0.001	51, (7.1)	34, (20.9)	<0.001	37, (8.5)	48, (10.8)	0.243
Daily drinking habits		139, (15.8)	47, (7.7)	92, (34.3)	<0.001	122, (17.1)	17, (10.4)	0.036	81, (18.6)	58, (13.1)	0.025
Smoking habit	Never smoked	617, (70.3)	528, (86.6)	89, (33.2)	<0.001	483, (67.6)	134, (82.2)	0.001	282, (64.8)	335, (75.6)	0.002
	Used to smoke	209, (23.8)	60, (9.8)	149, (55.6)		186, (26.0)	23, (14.1)		121, (27.8)	88, (19.9)	
	Smoking	52, (5.9)	22, (3.6)	30, (11.2)		46, (6.4)	6, (3.7)		32, (7.4)	20, (4.5)	
Living situation	Living alone	266, (30.3)	224, (36.7)	42, (15.7)	<0.001	202, (28.3)	64, (39.3)	0.006	119, (27.4)	147, (33.2)	0.060
Depression		159, (18.1)	124, (20.3)	35, (13.1)	0.010	111, (15.5)	48, (29.4)	<0.001	49, (11.3)	110, (24.8)	<0.001
Stroke		46, (5.2)	24, (3.9)	22, (8.2)	0.009	42, (5.9)	4, (2.5)	0.077	17, (3.9)	29, (6.5)	0.079
Heart disease		143, (16.3)	97, (15.9)	46, (17.2)	0.641	110, (15.4)	33, (20.2)	0.129	67, (15.4)	76, (17.2)	0.482
Diabetes		98, (11.2)	62, (10.2)	36, (13.4)	0.157	80, (11.2)	18, (11.0)	0.957	49, (11.3)	49, (11.1)	0.924
Cancer		128, (14.6)	91, (14.9)	37, (13.8)	0.667	102, (14.3)	26, (16.0)	0.582	67, (15.4)	61, (13.8)	0.493
Serum albumin	<4.0 g/dL	94, (10.7)	66, (10.8)	28, (10.4)	0.870	60, (8.4)	34, (20.9)	<0.001	38, (8.7)	56, (12.6)	0.061
Hemoglobin A1c	<6.0%	644, (73.3)	446, (73.1)	198, (73.9)	0.069	524, (73.3)	120, (73.6)	0.958	319, (73.3)	325, (73.4)	0.959
	6.0–6.4%	137, (15.6)	104, (17.0)	33, (12.3)		111, (15.5)	26, (16.0)		69, (15.9)	68, (15.3)	
	6.5%≤	97, (11.0)	60, (9.8)	37, (13.8)		80, (11.2)	17, (10.4)		47, (10.8)	50, (11.3)	

Statistical analysis was performed by chi-square test.

**Table 3:** Multivariable logistic regression analysis with sarcopenia as the dependent variable.

				95% Confidence Intervals	
Independent Variables		*p*-Value	Odds Ratio	Lower Limit	Upper Limit
Oral hypofunction	0:No, 1:Yes	0.041	1.585	1.019	2.465
Age	1-year increments	<0.001	1.111	1.075	1.148
Sex	0:Women, 1:Men	0.967	0.987	0.519	1.876
Body mass index	0:18.5≤, 1:<18.5	<0.001	4.036	2.248	7.246
Daily drinking habits	0:No, 1:Yes	0.746	1.114	0.579	2.146
Smoking habit	0:Never smoked	0.732	reference		
	1:Used to smoke	0.478	0.793	0.418	1.506
	2:Smoking	0.639	0.774	0.265	2.258
Living situation	0:Living with someone1:Living alone	0.733	1.078	0.701	1.658
Depression	0:No, 1:Yes	0.653	0.894	0.550	1.455
Education	1-year increments	0.083	0.933	0.863	1.009
JST-IC score	1-score increments	0.004	0.900	0.838	0.967
MMSE-J score	1-score increments	0.567	0.975	0.893	1.064
Stroke	0:No, 1:Yes	0.126	0.409	0.130	1.285
Heart disease	0:No, 1:Yes	0.493	1.196	0.716	1.998
Diabetes	0:No, 1:Yes	0.868	1.067	0.493	2.310
Cancer	0:No, 1:Yes	0.873	1.045	0.607	1.801
Serum albumin	0:4.0 g/dL≤, 1:<4.0 g/dL	0.013	1.995	1.157	3.439
Hemoglobin A1c	0:<6.0%	0.333	reference		
	1:6.0–6.4%	0.138	0.650	0.368	1.148
	2:6.5%≤	0.656	0.834	0.375	1.855
Constant		<0.001	0.001		

Abbreviations: JST-IC: Japan Science and Technology Agency Index of Competence; MMSE-J: Japanese version of the mini mental state examination; Q1: first quartile; Q3: third quartile; B: Partial regression coefficient

## Data Availability

The data that support the findings of this study are available from the corresponding author upon reasonable request. The data are not publicly available because of ethico-legal restrictions imposed by the Ethics Committee of the Tokyo Metropolitan Institute of Gerontology.
